# Phytochemical analysis and molecular docking studies of two endemic varieties of *Salvia sericeotomentosa*

**DOI:** 10.55730/1300-0527.3610

**Published:** 2023-09-30

**Authors:** Gülbahar Özge ALİM TORAMAN, Halil ŞENOL, Seçil YAZICI TÜTÜNİŞ, Nur TAN, Gülaçtı TOPÇU

**Affiliations:** 1 Department of Pharmacognosy, Faculty of Pharmacy, Bezmiâlem Vakıf University, İstanbul, Turkiye; 2 Department of Pharmacognosy, Faculty of Pharmacy, İstanbul University, İstanbul, Turkiye; 3 Department of Pharmacognosy, Institute of Health Sciences, İstanbul University, İstanbul, Turkiye; 4 Department of Pharmaceutical Chemistry, Faculty of Pharmacy, Bezmiâlem Vakıf University, İstanbul, Turkiye; 5 Center for Research and Practice in Drug Development from Natural Sources, İstanbul University, İstanbul, Turkiye; 6 Drug Application and Research Center (DARC), Bezmiâlem Vakif University, İstanbul, Turkiye

**Keywords:** *Salvia sericeotomentosa* var. *sericeotomentosa*, S*. sericeotomentosa* var. *hatayica*, LC-HRMS, antiinflammatory, molecular modeling

## Abstract

The use of medicinal plants for treating various diseases dates back thousands of years and has been a part of many cultures around the world. Various parts of plants, including roots, leaves, and flowers, and their extracts have been used to develop remedies to cure different ailments like fever, pain, inflammation, infections, among others. In this research, the aerial parts of both *Salvia* varieties were extracted with ethanol and water to obtain infusion and decoction, separately. S*. sericeotomentosa* var. *hatayica* Celep & Doğan (SH) and *Salvia sericeotomentosa* Rech. f. var. *sericeotomentosa* (ST) plants were chemically analyzed for polar compounds using LC-HRMS for the first time. All SH and ST extracts were found to be very rich in rosmarinic acid, salvianolic acid B, hispidulin-7-*O*-glucoside, and caffeic acid. The study also investigated the antiinflammatory and carbonic anhydrase inhibition properties of the most abundant secondary metabolites extracted from SH and ST. In silico studies were conducted for the first time to explore the effects of these metabolites on TNF-α, iNOS, and human carbonic anhydrase isoenzymes (hCAI and hCAII). Salvianolic acid B should be considered a strong antiinflammatory agent and a carbonic anhydrase I and II inhibitors due to low binding energy scores with the tested enzymes (TNF-α: −12.391 kcal/mol), (iNOS: −7.547 kcal/mol), (hCAI: −7.877 kcal/mol), and (hCAII: −4.312 kcal/mol).

## 1. Introduction

Medicinal plants have been used for treating diseases for centuries due to their therapeutic properties. According to research by the World Health Organization (WHO), a significant percentage of people (60–90%) in developed countries still prefer traditional herbal treatments because of their safety and efficacy [1]. Herbal medicines that are commonly used in traditional treatments come in various forms such as infusions, decoctions, tinctures, or plant extracts [2]. The genus *Salvia* comprises about 1000 species throughout the world, and its species commonly referred to “sage” which have been used in traditional folk medicine for centuries. Gene center of the genus *Salvia* is Anatolia and Asia, with more than 50% endemism ratio in Türkiye, belonging to Lamiaceae family [3]. *Salvia sericeotomentosa* has two varieties which are endemic to Türkiye. One of them is a novel variety *Salvia sericeotomentosa* var. *hatayica* that was identified by Celep et al. in 2009. They have white to cream corollas, a short glandular-hairy folium, and an inflorescence axis. The difference between them is that the calyx of ST is glabrous, while the calyx of SH is short glandular-hairy [4,5].

Recent bioactivity studies have shown that extracts and pure compounds of *Salvia* species possess multiple pharmacological effects, such as antimicrobial, antioxidative, antiinflammatory, hypoglycemic, cardiovascular, anxiolytic, antitumor, and sedative activities [3,6–11].

Inflammation is a natural process that occurs in response to injury or infection and involves a series of complex biological responses that include the activation of immune cells, the release of proinflammatory cytokines and other signaling molecules, and changes in blood vessel permeability. Nitric oxide, prostaglandin E2, and cytokine like tumor necrosis factor-alpha (TNF-alpha), interleukin-6, interleukin-1β play a vital role in the development of many inflammatory conditions [12,13]. Inducible nitric oxide synthase (iNOS) is an enzyme that produces nitric oxide (NO) in the body. Nitric oxide is involved in inflammation, which is the body’s immune response to harmful stimuli. While nitric oxide can have beneficial effects in regulating immune responses and blood flow, excessive or prolonged production of nitric oxide by iNOS can contribute to chronic inflammation and tissue damage. Therefore, the regulation of iNOS and nitric oxide production is important for maintaining the balance of inflammation in various diseases [14].

Carbonic anhydrase isoenzymes have many physiological processes, such as regulating acid-base balance in the body, transporting carbon dioxide in the blood, and secreting fluids in the digestive system. There are many different types of carbonic anhydrase enzymes; hCAI is primarily located in the cytoplasm of red blood cells and other tissues, while hCAII is found in various tissues, including the kidneys, lungs, and brain, involved in the reversible hydration of carbon dioxide, which is important for maintaining acid-base balance in the body [15].

Molecular docking investigates binding orientation and affinity of a ligand (a small molecule) to a protein receptor. The goal of docking is to identify the most favorable binding pose of the ligand within the protein’s binding site. Docking is used in drug discovery to screen large chemical libraries for potential drug candidates [16,17].

Secondary metabolites from medicinal plants, especially some *Salvia* species are rich sources of antiinflammatory agents [18–20]. In this study, the aerial parts of S*. sericeotomentosa* var. *hatayica* Celep & Doğan (SH) and *Salvia sericeotomentosa* Rech. f. var. *sericeotomentosa* (ST) species were subjected to chemical analysis for polar compounds by detecting LC-HRMS. The antiinflammatory and carbonic anhydrase inhibition activities of most abundant secondary metabolites of SH and ST extracts were investigated by in silico studies on TNF-α, iNOS, and human carbonic anhydrase isoenzymes (hCAI and hCAII) for the first time herein.

## 2. Materials and methods

### The plant material and extraction methods

The plant materials were collected from Arsuz-Hatay in May 2015. The voucher specimens, *Salvia sericeotomentosa* var. *hatayica* Celep & Doğan (SH) ISTE: 107535, and *Salvia sericeotomentosa* Rech. f. var. *sericeotomentosa* (ST) ISTE: 107536, were identified and placed in the Herbarium of the Faculty of Pharmacy, İstanbul University, Türkiye. The aerial parts of both *Salvia* varieties were air-dried and extracted with ethanol, and water to obtain infusion and decoction, separately. Therefore, three different extracts from each plant species were prepared to compare their secondary metabolites contents.

### LC-HRMS analysis

A previously established and validated LC-HRMS method was applied to determine the phenolic contents in the infusion, decoction, and ethanol extracts of *Salvia* species. LC and mass conditions (Tables S1 and S2) of the method were given in detail in supporting information [21]. The validation parameters of this study were linearity, recovery, repeatability, limits of the detection (LOD), and limits of the quantification (LOQ) (Tables S3–S5).

### Molecular docking studies

Molecular docking studies were performed to evaluate prospective interaction between compound and the target receptors. The inhibition profiles of the compounds against the TNF-α, iNOS, hCAI and hCAII enzymes were investigated by molecular docking. In silico studies were performed using Maestro 13.5 program of Schrödinger molecular modeling suite. Initially, the X-ray crystal structures of target proteins were obtained from RCSB Protein Data Bank: TNF-α (PDB ID: 7KP9 ), iNOS (PDB ID: 4NOS ), hCAI (PDB ID: 1BZM ), and hCAII (PDB ID: 1A42 ). Target compounds were drawn using Chem-Draw and transferred to Schrödinger for optimization studies using Maestro’s LigPrep software at physiological pH, and prepared possible stereoisomers and salts.

The preparation of receptors was conducted using Schrödinger’s Protein Preparation Wizard module. First, all the side chains, cocrystal ligands and water molecules were identified using the preprocess module. Then the main chain with the binding site was selected and other side chains and water molecules were deleted. Hydrogen bond optimization was performed at physiological pH value and the cocrystal in the binding site with the enzyme was minimized. The binding sites of each receptor were defined using Maestro’s receptor grid generation module. The site where the original cocrystal was located was defined as the binding site. By removing the cocrystal on each enzyme, a box containing the region was created for docking the target compounds to this region, and the protein was recorded as it is. Ligand-protein binding affinity was calculated using the MM-GBSA module. The binding interactions between each compound and the binding site of each receptor were analyzed individually. Finally, the compounds were docked collectively using Glide/XP [17,22,23].

## 3. Results

The two endemic varieties of *S. sericeotomentosa* species were investigated chemically by LC-HRMS and for antiinflammatory and carbonic anhydrase inhibition activities through in silico molecular docking studies for the first time in this study.

### Results of LC-HRMS analysis

LC-HRMS analysis results of polar extracts of both *S. sericeotomentosa* varieties (SH and ST) were given in Table 1 considering with their retention time. In an attempt to investigate polar compounds of SH and ST, ethanol extracts, and infusion and decoctions were prepared from each 100 g of the dried plants, and obtained extracts analyzed by LC-HRMS instrument, qualitatively and quantitatively. Among all six extracts, rosmarinic acid was found with the highest percentage which has strong antioxidant, cytotoxic, and antiinflammatory activities [24,25]. Following rosmarinic acid, salvianolic acid B, hispidulin-7-*O*-glucoside, caffeic acid, and homogentisic acid were found to be a remarkable amount in all six extracts.

Apigenin 7-*O*-acylglucoside, pinocembrin, genkwanin, vanillic acid, and p-coumaric acid were present only in ST and SH ethanol extracts while epigallocatechin gallate was present only in ST and SH water extracts (both in infusion and decoction). Luteolin-7-*O*-acylglucoside was present only in ST and SH infusion extracts. (-)-Rutin was not found SH decoction and ST infusion, also fairly low amount in the other extracts. Caffeine was observed in none of the extracts, except only in the SH ethanol extract with a very small amount (0.24 g/kg). There are ten glycosylated flavones and flavonols among all the detected polar compounds, but three of them, hispidulin-7-*O*-glucoside, luteolin-7-*O*-rutinoside, luteolin-7-*O*-glucoside were found to be high amount in the ethanol extracts, respectively.

### Molecular docking studies

According to LC-HRMS analysis the most abundant compounds were selected, and molecular docking studies were carried out to determine possible biological activity properties. For this purpose, the antiinflammatory and carbonic anhydrase inhibition activity of 9 compounds (Scheme) (caffeic acid, p-coumaric acid, rosmarinic acid, salvianolic acid B, hispidulin-7-*O*-glucoside, hispidulin, homogentisic acid, epigallocatechin, and chlorogenic acid) were evaluated as in silico methods on related proteins which are TNF-α, iNOS, hCAI, and hCAII. In silico studies, docking scores of selected compounds of ligand-protein complex were determined and given in Table 2. While aspirin and celecoxib were used as reference antiinflammatory drugs, acetazolamide was used as reference inhibitor of hCAI and hCAII.

According to the results of the molecular docking studies salvianolic acid B has the highest binding scores against TNF-α (–12.391 kcal/mol), iNOS (–17.547 kcal/mol), and hCAII (–14.312 kcal/mol). It is noteworthy that salvianolic acid B showed better activity than reference drugs used in this study. In addition to salvianolic acid B, hispidulin-7-*O*-glucoside and caffeic acid were found as the most active antiinflammatory compounds. Rosmarinic acid exhibited the highest binding score on hCAI enzyme while salvianolic acid B showed highest binding score on hCAII. On the other hand, caffeic acid showed moderate inhibition activity against hCAII. Among all docked compounds p-coumaric acid showed the worst binding score.

**Figure 1:**
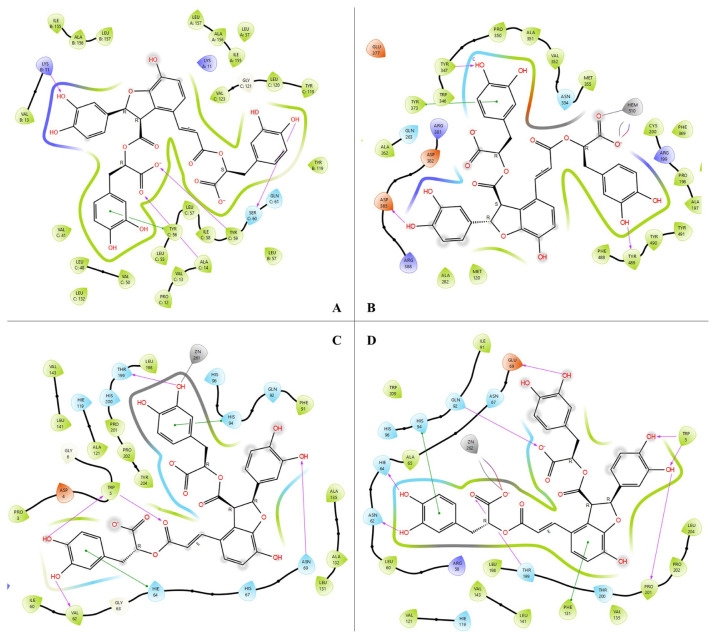
Molecular docking ligand-protein interactions between salvianolic acid B and active site of TNF-α (A), iNOS (B), hCAI (C), and hCAII (D).

Molecular docking ligand-protein interactions between salvianolic acid B and active site of TNF-α, iNOS, hCAI and hCAII were shown in Figure 1.

As can be seen from Figure 1A, salvianolic acid B formed four different hydrogen bond interactions (purple arrows) with amino acid residues which are Lys-11, Tyr-56, Tyr-59, and Ser-60. In addition, salvianolic acid B formed a π-π stacking interaction with Tyr-56 (green line). As seen in Figure 1B, three hydrogen bond interactions (purple arrows) are formed between salvianolic acid B and amino acid residues, including Tyr-347, Asp-385, and Tyr-489, in the active site of iNOS. Salvianolic acid B also interacts with iron atom of hemoglobin via carboxylate anion and formed a salt bridge complex. In addition, there is a π-π stacking interaction between Tyr-373 and benzene ring. As can be seen from the C part of Figure 1C, salvianolic acid B formed five different hydrogen bond interactions with Trp-5, Val-62, Asn-69, and Thr-199 which are the residue of binding site of hCAI. Salvianolic acid B also formed two different π-π stacking interactions with His-64 and His-94. In addition, salvianolic acid B interacted with Zn-261 residue of hCAI. This interaction showed that carbonic anhydrase can be easily inhibited by salvianolic acid B because hCAI needs Zn atom to activate. If Zn formed an interaction with any other molecule enzyme is not active [15]. In Figure 1D, salvianolic acid B interacted with Zn-262 and there are eight different hydrogen bond interactions between salvianolic acid B and Trp-5, Pro-201, Thr-199, Asn-62, His-64, His-94, Gln-92, ad Glu-69. These hydrogen bonds and metal-ligand interactions showed that salvianolic acid B can bind as strongly to active site of hCAII and inhibit it. After molecular docking studies to determine free binding energies of ligand protein complexes, the MM-GBSA ΔG binding energies were calculated by Schrödinger Prime MM-GBSA module. The MM-GBSA ΔG binding energies of salvianolic acid B-TNF-α, salvianolic acid B-iNOS, salvianolic acid B-hCAI, and salvianolic acid B-hCAII complexes were determined as −36.30, −53.85, −24.52 and −53.85 kcal/mol, respectively. Docking scores are compatible with MM-GBSA ΔG binding energies. The smaller the free binding energy, the more affinity ligands bind to proteins. Salvianolic acid B has very high docking scores, especially against iNOS (–17.547 kcal/mol) and hCAII (–14.312 kcal/mol) and can easily interact with enzymes thanks to its low binding energies. These results suggest that salvianolic acid B can be both an antiinflammatory and a carbonic anhydrase II inhibitor.

**Figure 2:**
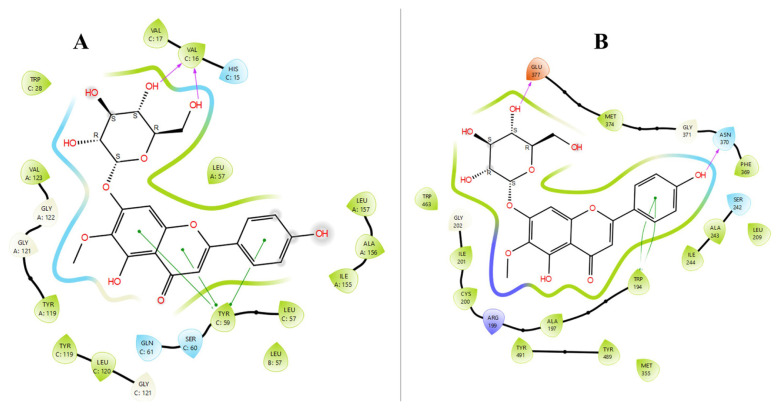
Molecular docking ligand-protein interactions between hispidulin-7-*O*-glucoside and active site of TNF-α (A) and iNOS (B).

According to the molecular docking studies, in addition to salvianolic acid B, hispidulin-7-*O*-glucoside and caffeic acid showed the best activity against TNF-α and iNOS as potential antiinflammatory agents. According to the docking scores, for antiinflammatory activity hispidulin-7-*O*-glucoside showed a strong binding effect and has high binding scores on both TNF-α (–11.473 kcal/mol) and iNOS (–10.167 kcal/mol), while caffeic acid was only active against iNOS (–11.395 kcal/mol). Furthermore, caffeic acid has a high binding score against hCAII (–8.047 kcal/mol). Molecular docking ligand-protein interactions between hispidulin-7-*O*-glucoside and active sites of TNF-α and iNOS were given in Figure 2 as combined.

As can be seen from Figure 2A, hispidulin-7-*O*-glucoside formed two different hydrogen bond interactions and three different π-π stacking interactions with amino acid residue of active site of TNF-α. The MM-GBSA ΔG binding free energy of hispidulin-7-*O*-glucoside -TNF-α complex was found as −55.10 kcal/mol. In Figure 2B, hispidulin-7-*O*-glucoside formed two different hydrogen bond interactions with Glu-377 and Asn-370 of active site of iNOS. Also, hispidulin-7-*O*-glucoside formed π-π stacking interactions with Trp-194. The MM-GBSA ΔG binding free energy of hispidulin-7-*O*-glucoside -iNOS complex was found as −37.67 kcal/mol.

**Figure 3:**
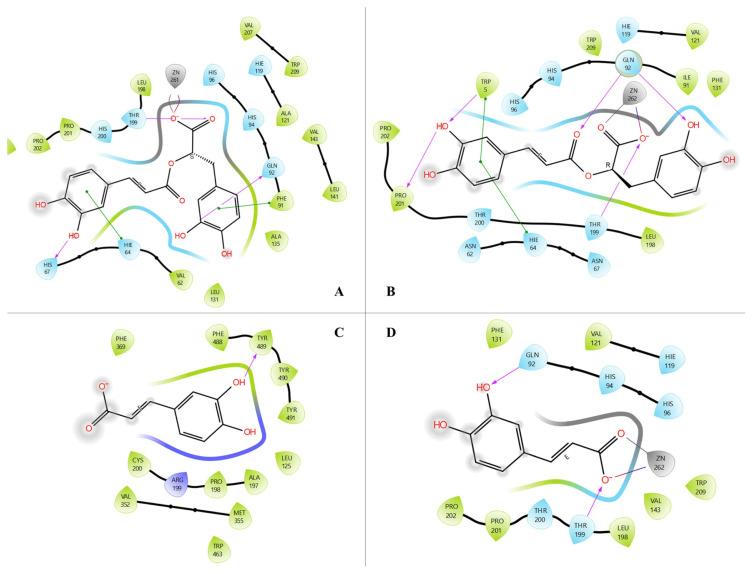
Molecular docking ligand-protein interactions between rosmarinic acid and active site of hCAI (A) and hCAII (B) and ligand-protein interactions between caffeic acid and active site of iNOS (C) and hCAII (D).

Caffeic acid showed both iNOS and hCAII inhibition activity as in silico with docking scores −11.395 and −8.047 kcal/mol, respectively. According to molecular docking studies besides to salvianolic acid B, rosmarinic acid was found as one of the most active inhibitors of both hCAI and hCAII. Molecular docking ligand-protein interactions between rosmarinic acid and active sites of hCAI and hCAII were given in Figure 3 as combined. Figure 3 also contains ligand-protein interactions between caffeic acid and active sites of iNOS and hCAII.

As can be seen from Figure 3A, rosmarinic acid interacted with Zn-261 in the active site of the hCAI enzyme. In addition, it formed three different hydrogen bonds (purple arrows) with His-67, Gln-92, Thr-199 in the active site of hCAI. The interaction of zinc in the active site of the enzyme with the inhibitor is very important for the inhibition of this enzyme. The MM-GBSA ΔG free binding energy of the rosmarinic acid -hCAI complex was found to be −23.10 kcal/mol. Figure 3B shows the interactions of rosmarinic acid with the active site of hCAII. Rosmarinic acid formed five different hydrogen bond interactions with four different amino acid residues (Trp-5, Pro-201, Gln-92, Thr-199) in the active site of hCAII. In addition, Zn-262 in the active site of the enzyme formed a complex with the carboxylic acid group of the rosmarinic acid via a salt bridge. Just like in hCAI, the interaction of zinc with the inhibitor is very important for the inhibition of the enzyme. Caffeic acid had a high binding score only for iNOS (–11.395 kcal/mol) and hCAII (–8.047 kcal/mol) in this study. In Figure 3C, caffeic acid interacts with the Tyr-489 amino acid residue of iNOS via hydrogen bond interaction. Figure 3D shows the interactions of caffeic acid with hCAII. Caffeic acid interacted with Gln-92 and Thr-199 in the active site of hCAII via hydrogen bond interactions and it interacted with Zn-262 through the salt bridge. The MM-GBSA ΔG free binding energies of caffeic acid -iNOS and caffeic acid-hCAII complexes were found as −34.20 and −8.33 kcal/mol, respectively.

**Figure 4:**
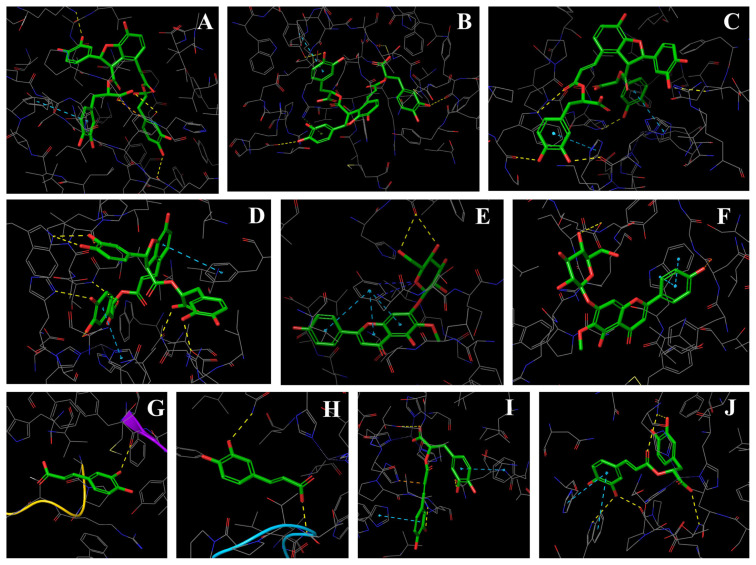
Molecular docking 3D interactions: ***A)*** salvianolic acid B-TNF-α, ***B)*** salvianolic acid B-iNOS, ***C)*** salvianolic acid B-hCAI, ***D)*** salvianolic acid B -hCAII, ***E)*** hispidulin-7-*O*-glucoside -TNF-α, ***F)*** hispidulin-7-*O*-glucoside -iNOS, ***G)*** caffeic acid-iNOS, ***H)*** caffeic acid-hCAII, ***I)*** rosmarinic acid -hCAI, and ***J)*** rosmarinic acid -hCAII complexes.

When evaluate the docking scores of other molecules, epigallocatechin and chlorogenic acid also have high binding scores against all four enzymes, but their binding scores are lower than the binding scores of salvianolic acid B, hispidulin-7-*O*-glucoside, and caffeic acid. When the results were compared with the antiinflammatory drugs aspirin and celecoxib, it was seen that the binding scores of salvianolic acid B, hispidulin-7-*O*-glucoside and caffeic acid were much higher than the reference drugs and they stand out as the first three molecules. According to the results obtained from in silico carbonic anhydrase inhibition activity studies, rosmarinic acid has the highest binding scores against hCAI and salvianolic acid B has the highest binding scores against hCAII. In addition, caffeic acid showed an inhibitory effect on hCAII almost as much as rosmarinic acid. Molecular docking 3D ligand protein interactions of all 2D images were given in Figure 4 as combined.

In Figure 4A–4J, the hydrogen bonds were presented by yellow dashes, the π-π stacking interactions were presented by turquois dashes, the π-cation interactions were presented by green dashes, and the hydrophobic contacts were presented by orange dashes.

### Molecular docking validation studies

The cocrystallized ligands of TNF-α **(**7KP9-A7G), iNOS (4NOS-H2B), hCA-I (1BZM-MZM) and hCA-II (1A42-BZU) were redocked at their actual crystal positions without changing their states or producing any conformers, thereby validating the molecular docking methods and protocols [17]. The original crystallographic conformation was superimposed with the cocrystallized ligand’s docked pose, and the RMSD values were found to be 2.1984 Å for 7KP9, 1.3065 Å for 4NOS, 1.7528 Å for 1BZM and 1.1704 Å for 1A42. RMSD (root mean square deviation) values are often used to determine the quality of reproductive binding pose by molecular docking. The poses with RMSD less than 2 Å are often used as a criterion of the correct bound structure prediction while the value between 2 and 3 Å is acceptable [23,26,27].

## 4. Discussion

The water and ethanol extracts of Salvia sericeotomentosa species were detected by LC-HRMS for the first time in this study, SH and ST polar extracts were found to be very rich in three phenolic acids (rosmarinic acid, salvianolic acid B, and caffeic acid) and a flavone glycoside hispidulin-7-*O*-glucoside.

According to recent studies, carbonic anhydrase inhibitors may have an impact on inflammation [15]. Due to relationships inflammation and carbonic anhydrase, they were evaluated together in this study. Based on the results of LC-HRMS analysis of the six extracts, the compounds detected only in high amount were investigated by in silico tests.

Caffeic acid, hispidulin-7-*O*-glucoside, and salvianolic acid B were identified as promising antiinflammatory compounds based on the outcomes of in silico research. On the other hand, salvianolic acid B, rosmarinic acid, and caffeic acid were found as potential hCAI and hCAII inhibitors. According to in silico studies, salvianolic acid B is both antiinflammatory agent and hCAII inhibitor; hispidulin-7-*O*-glucoside has a potential use as an antiinflammatory agent while rosmarinic acid has an hCAII inhibitory activity.

Regarding the low to binding scores of salvianolic acid B with relevant enzymes (TNF-α: −12.391 kcal/mol; iNOS: −17.547 kcal/mol; hCAI: −7.877 kcal/mol, and hCAII: −14.312 kcal/mol), it can be easily understood that salvianolic acid B is the strongest antiinflammatory agent and a carbonic anhydrase inhibitor among the tested compounds. Therefore, salvianolic acid B could be further investigated for its antiinflammatory and carbonic anhydrase inhibition activity properties by in vitro and in vivo studies. If the compound is proved to be effective and safe, it could be developed into a new inflammatory treatment option.

As a conclusion, both varieties; *Salvia sericeotomentosa* Rech. f. var. *sericeotomentosa* (ST) and S*. sericeotomentosa* var. *hatayica* Celep & Doğan (SH) must be investigated as potential source in finding new antiinflammatory and carbonic anhydrase inhibitors as well as anticancer agents. In addition, isolation studies of pure compounds from nonpolar extracts are continuing to catch druggable compounds which are responsible for the inflammatory effects.

## Supporting information

**Phytochemical analysis and molecular docking studies of two endemic varieties of** ***Salvia sericeotomentosa***

**Table S1:** The mobile phase with gradient program of SH and ST extracts.

Gradient time	Flow (mL/min)	% A (1% formic acid-H _2_O)	%B (1% formic acid-MeOH)
0.00	0.35	50	50
1.00	0.35	50	50
3.00	0.35	0	100
6.00	0.35	0	100
7.00	0.35	50	50
15.00	0.35	50	50

**Table S2:** MS conditions (mass spectrometer) of SH and ST extracts of LC-HRMS analysis.

**System**	Thermo Orbitrap Q-exactive
**Ion source**	ESI
**Column**	Troyasil C18 HS − 150 × 3 mm 5 μ
**Mass scanning range**	100–900 m/z
**Sheath gas flow rate**	45
**Aux gas flow rate**	10
**Spray voltage (kV)**	3.80a
**Capillary temp. (°C)**	320
**Aux gas heater temp. (°C)**	320
**S-lens RF level**	50.0
**Definitions**	ILMER Library

**Table S3:** Analytical parameters of LC-MS/MS methods of ethanol extracts.

Compounds	ST	SH	Relative uncertainty (%)	Molecular formula	m/z	Ion mode	Linear range	Equation	LOD/LOQ	R ^2^	Recovery	%RSD
**Ascorbic acid**	393.50	598.48	3.94	C _6_H _8_O _6_	175.02	Negative	0.5–10	y = 0.00347x − 0.00137	0.39/1.29	0.9988	96.2	2.93
**(-)-Epigallocatechin**	300.55	508.75	3.09	C _15_H _14_O _7_	307.08	Positive	0.3–5	y = 0.00317x + 0.000443	0.17/0.57	0.9947	102.22	3.18
**Chlorogenic acid**	249.51	135.84	3.58	C _16_H _18_O _9_	353.09	Negative	0.05–10	y = 0.00817x + 0.000163	0.02/0.06	0.9994	96.68	3.93
**Verbascoside**	161.64	146.72	2.93	C _29_H _36_O _15_	623.20	Negative	0.1–10	y = 0.00758x + 0.000563	0.03/0.1	0.9995	96.19	3.02
**Orientin**	16.91	139.26	3.67	C _21_H _20_O _11_	447.09	Negative	0.1–10	y = 0.00757x + 0.000347	0.01/0.03	0.9993	96.22	4.16
**Caffeic acid**	3784.07	4560.78	3.74	C _9_H _8_O _4_	179.03	Negative	0.3–10	y = 0.0304x + 0.00366	0.08/0.27	0.9993	94.51	3.23
**Caffeine**		0.24	3.06	C _8_H _10_N _4_O _2_	195.09	Positive	0.05–7	y = 0.122x + 0.00366	0.01/0.03	0.9987	92.89	3.65
**(+)-t** ***rans*** ** taxifolin**	59.73	88.01	3.35	C _15_H _12_O _7_	303.05	Negative	0.3–10	y = 0.0289x + 0.00537	0.01/0.03	0.9978	91.66	3.26
**Luteolin-7-** ***O*** **-rutinoside**	815.81	932.03	3.06	C _27_H _30_O _15_	593.15	Negative	0.1–10	y = 0.00879x + 0.000739	0.01/0.03	0.9988	93.05	3.84
**Vanillic acid**	146.93	400.84	3.49	C _8_H _8_O _4_	167.03	Negative	0.3–10	y = 0.00133x + 0.0003456	0.1/0.33	0.9997	98.66	4.74
**Luteolin 7-** ***O*** **-glucoside**	512.52	615.14	4.14	C _21_H _20_O _11_	447.0933	Negative	0.1–7	y = 0.0162x + 0.00226	0.01/0.03	0.9961	96.31	3.56
**p-coumaric acid**	1173.23	1664.39	3.31	C _9_H _8_O _3_	163.04	Negative	1 + 10	y = 0.000324x − 0.0000641	0.32/1.02	0.9988	117.01	4.01
**Hesperidin**	84.34	42.33	3.79	C _28_H _34_O _15_	609.18	Negative	0.05–10	y = 0.00423x + 0.0000138	0.01/0.03	0.9994	96.14	3.23
**Rutin**	21.92	10.88	3.07	C _27_H _30_O _16_	609.15	Negative	0.05–10	y = 0.00329x − 0.00005576	0.01/0.03	0.999	96.97	4.12
**Rosmarinic acid**	58912.33	59015.24	3.77	C _18_H _16_O _8_	359.08	Negative	0.05–10	y = 0.00717x − 0.0003067	0.01/0.03	0.9992	99.85	4.16
**Hyperoside**	202.58	274.36	3.46	C _21_H _20_O _12_	463.09	Negative	0.05–10	y = 0.0072x − 0.00003096	0.01/0.03	0.9995	96.62	3.77
**Apigenin 7-** ***O*** **-glucoside**	312.33	271.89	3.59	C _21_H _20_O _10_	431.10	Negative	0.3–7	y = 0.0246x + 0.00306	0.01/0.03	0.9962	96.07	4.61
**Nepetin-7-** ***O*** **-glucoside**	182.78	179.97	3.07	C _22_H _22_O _12_	479.12	Positive	0.05–10	y = 0.00629x − 0.0001951	0.01/0.03	0.9997	102.18	3.51
**Quercetin**	3.99	9.70	2.95	C _15_H _10_O _7_	301.04	Negative	0.1–10	y = 0.0509x + 0.00467	0.01/0.03	0.9978	96.41	2.90
**Salicylic acid**	651.19	823.34	1.89	C _7_H _6_O _3_	137.02	Negative	0.3–10	y = 0.0361x + 0.00245	0.01/0.03	0.9982	92.88	3.97
**Naringenin**	11.23	16.66	4.20	C _15_H _12_O _5_	271.06	Negative	0.1–10	y = 0.0281x + 0.00182	0.01/0.03	0.9995	86.65	1.52
**Luteolin**	175.15	172.33	3.42	C _15_H _10_O _6_	285.04	Negative	0.1–10	y = 0.117x + 0.00848	0.01/0.03	0.9981	96.98	4.77
**Nepetin**	66.54	79.19	2.19	C _16_H _12_O7	315.05	Negative	0.05–10	y = 0.0853x + 0.00269	0.01/0.03	0.9992	97.76	3.70
**Apigenin**	332.92	236.93	2.87	C _15_H _10_O _5_	269.05	Negative	0.3–10	y = 0.104x + 0.0199	0.01/0.03	0.9998	81.55	4.07
**Hispidulin**	258.04	377.60	3.41	C _16_H _12_O _6_	301.07	Positive	0.05–10	y = 0.02614x + 0.0003114	0.01/0.03	0.9993	98.36	2.91
**Caffeic acid phenethyl ester**	0.55	1.01	3.13	C _17_H _16_O _4_	283.10	Negative	0.3–7	y = 0.255x + 0.0477	0.01/0.03	0.9964	94.42	3.66
**Chrysin**	1.41	1.32	3.24	C _15_H _10_O _4_	253.05	Negative	0.05–7	y = 0.0964x − 0.0002622	0.01/0.03	0.999	87.92	2.36
**Acacetin**	13.74	30.71	3.98	C _16_H _12_O _5_	283.06	Negative	0.05–7	y = 0.046x + 0.0001875	0.01/0.03	0.9995	87.52	3.55
**Dihydrocaffeic acid**	21.53	35.14	0.86	C _18_H _23_NO _7_	366.15473	Negative	0.5–10	y = 0.06102x − 0.00989	0.14/0.46	0.999	100.77	1.05
**6-OH-luteolin-7-** ***O*** **-glucoside**	14.05	14.39	2.99	C _21_H _20_O _12_	463.0882	Negative	0.5–10	y = 0.01482x − 0.001803	0.23/0.77	0.997	102.44	3.66
**6-O-Me-luteolin-7-** ***O*** **-glucoside**	130.37	89.43	2.58	C _21_H _20_O _12_	477.10449	Negative	0.5–10	y = 0.02636x − 0.00254	0.18/0.61	0.9982	101.21	3.16
**Apigenin 7-** ***O*** **-acylglucoside**	48.57	20.17	2.70	C _22_H _30_O _11_	473.10947	Negative	0.5–10	y = 0.01496x − 0.0006413	0.13/0.42	0.9986	99.12	3.31
**Chrysoeriol**	155.54	176.96	2.08	C _16_H _12_O _6_	299.05611	Negative	0.5–10	y = 0.1023x − 0.002224	0.15/0.5	0.9974	96.42	2.55
**Cirsilineol**	32.21	35.71	2.33		313.07227	Negative	0.5–10	y = 0.1389x + 0.02817	0.22/0.72	0.9955	96.17	2.86
**Apigenin 7-methylate**	1.84	4.05	2.94	C _15_H _12_O _5_	283.06149	Negative	0.5–10	y = 0.3563x	0.16/0.54	0.9949	97.1	3.60
**Sclareol**	1.72	5.37	3.96	C _20_H _36_O _2_	273.25745	Positive	0.5–10	y = 0.3233x + 0.0004172	0.12/0.39	0.9984	100.59	4.85
**Homogentisic acid**	929.78	1331.28	4.35	C _8_H _8_O _4_	167.03498	Negative	0.5–10	y = 0.01076x − 0.002027	0.13/0.45	0.9987	100.76	5.33
**3,4-dihydroxy benzaldehyde**	164.27	223.31	3.79	C _7_H _6_O _3_	137.02442	Negative	0.5–10	y = 1.343x + 0.6441	0.53/1.77	0.9962	98.67	4.64
**2,5-dihydroxybenzoic acid**	33.03	30.95	4.77	C _7_H _6_O _4_	153.01933	Negative	0.5–10	y = 0.06114x − 0.0102	0.17/0.57	0.9985	100.23	5.84
**Salvianolic acid B**	14911.62	13111.55	6.50	C _36_H _30_O _16_	717.14611	Negative	1–10	y = 0.0005342x − 0.0003522	0.33/1.1	0.9989	98.96	7.96
**Hispidulin 7-** ***O*** **-glucoside**	26517.26	26709.56	4.57	C _22_H _22_O _11_	461.10893	Negative	0.5–10	y = 0.0003758x-0.00006441	0.57/1.91	0.9988	101.76	5.60
**Pinocembrin**	0.39	0.34	3.28	C _15_H _12_O _4_	255.06628	Negative	0.5–10	y = 0.5224x	0.17/0.57	0.9971	101.89	4.02
**Genkwanin**	0.04	1.72	4.44	C _16_H _12_O _5_	283.0612	Negative	0.5–10	y = 0.4311x + 0.01952	0.16/0.54	0.9985	100.44	5.44
**Carnosic acid**	1.96	0.03	2.58	C _20_H _28_O _4_	331.19148	Negative	0.5–10	y = 0.342x + 0.0485	0.21/0.69	0.9984	100.14	3.16

R^2^: coefficient of determination; LOD/LOQ (mg/L): limit of detection/quantification.

**Table S4:** Analytical parameters of LC-HRMS methods of Infusion.

Compounds	ST	SH	Relative uncertainty (%)	Molecular formula	m/z	Ion mode	Linear range	Equation	LOD/LOQ	R ^2^	Recovery	%RSD
**(-)-Epigallocatechin**	293.28	211.15	3.09	C _15_H _14_O _7_	307.0812	Positive	0.3–5	y = 0.00317x + 0.000443	0.17/0.57	0.9947	102.22	3.18
**(-)-Epigallocatechin gallate**	10.06	4.25	3.76	C _22_H _18_O _11_	459.0922	Positive	0.3–7	y = 0.00182x + 0.000026	0.1/0.33	0.9989	94.76	4.20
**Chlorogenic acid**	305.49	131.31	3.58	C _16_H _18_O _9_	353.0878	Negative	0.05–10	y = 0.00817x + 0.000163	0.02/0.06	0.9994	96.68	3.93
**Verbascoside**	11.29	10.95	2.93	C _29_H _36_O _15_	623.1981	Negative	0.1–10	y = 0.00758x + 0.000563	0.03/0.1	0.9995	96.19	3.02
**Orientin**	1.59	8.21	3.67	C _21_H _20_O _11_	447.0933	Negative	0.1–10	y = 0.00757x + 0.000347	0.01/0.03	0.9993	96.22	4.16
**Caffeic acid**	2453.24	1750.28	3.74	C _9_H _8_O _4_	179.0350	Negative	0.3–10	y = 0.0304x + 0.00366	0.08/0.27	0.9993	94.51	3.23
**(+)-t** ***rans*** ** taxifolin**	1.71	2.94	3.35	C _15_H _12_O _7_	303.0510	Negative	0.3–10	y = 0.0289x + 0.00537	0.01/0.03	0.9978	91.66	3.26
**Luteolin-7-** ***O*** **-rutinoside**	87.63	121.03	3.06	C _27_H _30_O _15_	593.1512	Negative	0.1–10	y = 0.00879x + 0.000739	0.01/0.03	0.9988	93.05	3.84
**Luteolin 7-** ***O*** **-glucoside**	28.23	34.76	4.14	C _21_H _20_O _11_	447.0933	Negative	0.1–7	y = 0.0162x + 0.00226	0.01/0.03	0.9961	96.31	3.56
**Hesperidin**	11.41	6.98	3.79	C _28_H _34_O _15_	609.1825	Negative	0.05–10	y = 0.00423x + 0.0000138	0.01/0.03	0.9994	96.14	3.23
**Rutin**		0.83	3.07	C _27_H _30_O _16_	609.1461	Negative	0.05–10	y = 0.00329x − 0.00005576	0.01/0.03	0.999	96.97	4.12
**Rosmarinic acid**	30283.78	21426.51	3.77	C _18_H _16_O _8_	359.0772	Negative	0.05–10	y = 0.00717x − 0.0003067	0.01/0.03	0.9992	99.85	4.16
**Hyperoside**	21.71	16.90	3.46	C _21_H _20_O _12_	463.0882	Negative	0.05–10	y = 0.0072x − 0.00003096	0.01/0.03	0.9995	96.62	3.77
**Apigenin-7-** ***O*** **-glucoside**	9.62	7.78	3.59	C _21_H _20_O _10_	431.0984	Negative	0.3–7	y = 0.0246x + 0.00306	0.01/0.03	0.9962	96.07	4.61
**Nepetin-7-** ***O*** **-glucoside**	85.57	45.40	3.07	C _22_H _22_O _12_	479.1184	Positive	0.05–10	y = 0.00629x − 0.0001951	0.01/0.03	0.9997	102.18	3.51
**Quercetin**	0.48	0.52	2.95	C _15_H _10_O _7_	301.0354	Negative	0.1–10	y = 0.0509x + 0.00467	0.01/0.03	0.9978	96.41	2.90
**Salicylic acid**	162.03	127.42	1.89	C _7_H _6_O _3_	137.0244	Negative	0.3–10	y = 0.0361x + 0.00245	0.01/0.03	0.9982	92.88	3.97
**Naringenin**	1.59	1.23	4.20	C _15_H _12_O _5_	271.0612	Negative	0.1–10	y = 0.0281x + 0.00182	0.01/0.03	0.9995	86.65	1.52
**Luteolin**	38.97	24.68	3.42	C _15_H _10_O _6_	285.0405	Negative	0.1–10	y = 0.117x + 0.00848	0.01/0.03	0.9981	96.98	4.77
**Nepetin**	8.51	5.04	2.19	C _16_H _12_O7	315.0510	Negative	0.05–10	y = 0.0853x + 0.00269	0.01/0.03	0.9992	97.76	3.70
**Apigenin**	18.49	13.02	2.87	C _15_H _10_O _5_	269.0456	Negative	0.3–10	y = 0.104x + 0.0199	0.01/0.03	0.9998	81.55	4.07
**Hispidulin**		24.29	3.41	C _16_H _12_O _6_	301.0707	Positive	0.05–10	y = 0.02614x + 0.0003114	0.01/0.03	0.9993	98.36	2.91
**Caffeic acid phenethyl ester**	0.12	0.04	3.13	C _17_H _16_O _4_	283.0976	Negative	0.3–7	y = 0.255x + 0.0477	0.01/0.03	0.9964	94.42	3.66
**Chrysin**	0.87	0.67	3.24	C _15_H _10_O _4_	253.0506	Negative	0.05–7	y = 0.0964x − 0.0002622	0.01/0.03	0.999	87.92	2.36
**Acacetin**	2.54	2.66	3.98	C _16_H _12_O _5_	283.0612	Negative	0.05–7	y = 0.046x + 0.0001875	0.01/0.03	0.9995	87.52	3.55
**Dihydrocaffeic acid**	75.90	72.94	0.86	C _18_H _23_NO _7_	366.15473	Negative	0.5–10	y = 0.06102x − 0.00989	0.14/0.46	0.999	100.77	1.05
**6-OH-luteolin-7-O-glucoside**	16.06	13.89	2.99	C _21_H _20_O _12_	463.0882	Negative	0.5–10	y = 0.01482x − 0.001803	0.23/0.77	0.997	102.44	3.66
**6-O-Me-luteolin-7-O-glucoside**	98.37	52.50	2.58	C _21_H _20_O _12_	477.10449	Negative	0.5–10	y = 0.02636x − 0.00254	0.18/0.61	0.9982	101.21	3.16
**Luteolin-7-O-acylglucoside**	174.99	141.79	2.12	C _22_H _20_O _12_	475.0882	Negative	0.5–10	y = 0.003676x + 0.0002104	0.27/0.89	0.9964	98.03	2.59
**Chrysoeriol**	18.65	14.48	2.08	C _16_H _12_O _6_	299.05611	Negative	0.5–10	y = 0.1023x − 0.002224	0.15/0.5	0.9974	96.42	2.55
**Apigenin 7-methylate**	0.36	0.36	2.94	C _15_H _12_O _5_	283.06149	Negative	0.5–10	y = 0.3563x	0.16/0.54	0.9949	97.1	3.60
**Sclareol**	0.72	0.36	3.96	C _20_H _36_O _2_	273.25745	Positive	0.5–10	y = 0.3233x + 0.0004172	0.12/0.39	0.9984	100.59	4.85
**Homogentisic acid**	779.92	639.56	4.35	C _8_H _8_O _4_	167.03498	Negative	0.5–10	y = 0.01076x − 0.002027	0.13/0.45	0.9987	100.76	5.33
**3,4-dihydroxybenzaldehyde**	30.18	15.24	3.79	C _7_H _6_O _3_	137.02442	Negative	0.5–10	y = 1.343x + 0.6441	0.53/1.77	0.9962	98.67	4.64
**2,5-dihydroxybenzoic acid**	20.87	23.81	4.77	C _7_H _6_O _4_	153.01933	Negative	0.5–10	y = 0.06114x − 0.0102	0.17/0.57	0.9985	100.23	5.84
**Salvianolic acid B**	3458.41	2785.28	6.50	C _36_H _30_O _16_	717.14611	Negative	1–10–	y = 0.0005342x − 0.0003522	0.33/1.1	0.9989	98.96	7.96
**Hispidulin 7-** ***O*** **-glucoside**	1976.26	1796.51	4.57	C _22_H _22_O _11_	461.10893	Negative	0.5–10	y = 0.0003758x − 0.00006441	0.57/1.91	0.9988	101.76	5.60
**Carnosic acid**	0.32	0.32	2.58	C _20_H _28_O _4_	331.19148	Negative	0.5–10	y = 0.342x + 0.0485	0.21/0.69	0.9984	100.14	3.16

R^2^: coefficient of determination; LOD/LOQ (mg/L): limit of detection/quantification.

**Table S5:** Analytical parameters of LC-HRMS methods of decoction extracts.

Compounds	ST	SH	Relative uncertainty (%)	Molecular formula	m/z	Ion mode	Linear range	Equation	LOD/LOQ	R ^2^	Recovery	%RSD
**Ascorbic acid**	273.18	333.73	3.94	C _6_H _8_O _6_	175.0248	Negative	0.5–10	y = 0.00347x − 0.00137	0.39/1.29	0.9988	96.2	2.93
**(-)-Epigallocatechin**	538.47	615.99	3.09	C _15_H _14_O _7_	307.0812	Positive	0.3–5	y = 0.00317x + 0.000443	0.17/0.57	0.9947	102.22	3.18
**(-)-Epigallocatechin gallate**	12.57	5.16	3.76	C _22_H _18_O _11_	459.0922	Positive	0.3–7	y = 0.00182x + 0.000026	0.1/0.33	0.9989	94.76	4.20
**Chlorogenic acid**	824.02	540.08	3.58	C _16_H _18_O _9_	353.0878	Negative	0.05–10	y = 0.00817x + 0.000163	0.02/0.06	0.9994	96.68	3.93
**Verbascoside**	41.49	38.89	2.93	C _29_H _36_O _15_	623.1981	Negative	0.1–10	y = 0.00758x + 0.000563	0.03/0.1	0.9995	96.19	3.02
**Orientin**		23.02	3.67	C _21_H _20_O _11_	447.0933	Negative	0.1–10	y = 0.00757x + 0.000347	0.01/0.03	0.9993	96.22	4.16
**Caffeic acid**	5446.51	6159.05	3.74	C _9_H _8_O _4_	179.0350	Negative	0.3–10	y = 0.0304x + 0.00366	0.08/0.27	0.9993	94.51	3.23
**(+)-t** ***rans*** ** taxifolin**	5.90	6.11	3.35	C _15_H _12_O _7_	303.0510	Negative	0.3–10	y = 0.0289x + 0.00537	0.01/0.03	0.9978	91.66	3.26
**Luteolin-7-** ***O*** **-rutinoside**	244.06	267.58	3.06	C _27_H _30_O _15_	593.1512	Negative	0.1–10	y = 0.00879x + 0.000739	0.01/0.03	0.9988	93.05	3.84
**Luteolin-7-** ***O*** **-glucoside**	119.20	111.90	4.14	C _21_H _20_O _11_	447.0933	Negative	0.1–7	y = 0.0162x + 0.00226	0.01/0.03	0.9961	96.31	3.56
**Hesperidin**	21.65	11.75	3.79	C _28_H _34_O _15_	609.1825	Negative	0.05–10	y = 0.00423x + 0.0000138	0.01/0.03	0.9994	96.14	3.23
**Rutin**	1.49		3.07	C _27_H _30_O _16_	609.1461	Negative	0.05–10	y = 0.00329x − 0.00005576	0.01/0.03	0.999	96.97	4.12
**Rosmarinic acid**	47396.59	48091.43	3.77	C _18_H _16_O _8_	359.0772	Negative	0.05–10	y = 0.00717x − 0.0003067	0.01/0.03	0.9992	99.85	4.16
**Hyperoside**	38.93	51.31	3.46	C _21_H _20_O _12_	463.0882	Negative	0.05–10	y = 0.0072x − 0.00003096	0.01/0.03	0.9995	96.62	3.77
**Apigenin -7-** ***O*** **-glucoside**	33.30	25.67	3.59	C _21_H _20_O _10_	431.0984	Negative	0.3–7	y = 0.0246x + 0.00306	0.01/0.03	0.9962	96.07	4.61
**Nepetin-7-** ***O*** **-glucoside**	157.78	116.31	3.07	C _22_H _22_O _12_	479.1184	Positive	0.05–10	y = 0.00629x − 0.0001951	0.01/0.03	0.9997	102.18	3.51
**Quercetin**	2.30	2.66	2.95	C _15_H _10_O _7_	301.0354	Negative	0.1–10	y = 0.0509x + 0.00467	0.01/0.03	0.9978	96.41	2.90
**Salicylic acid**	243.56	285.99	1.89	C _7_H _6_O _3_	137.0244	Negative	0.3–10	y = 0.0361x + 0.00245	0.01/0.03	0.9982	92.88	3.97
**Naringenin**	0.73	1.31	4.20	C _15_H _12_O _5_	271.0612	Negative	0.1–10	y = 0.0281x + 0.00182	0.01/0.03	0.9995	86.65	1.52
**Luteolin**	38.47	57.82	3.42	C _15_H _10_O _6_	285.0405	Negative	0.1–10	y = 0.117x + 0.00848	0.01/0.03	0.9981	96.98	4.77
**Nepetin**	6.63	9.01	2.19	C _16_H _12_O7	315.0510	Negative	0.05–10	y = 0.0853x + 0.00269	0.01/0.03	0.9992	97.76	3.70
**Apigenin**	24.37	26.63	2.87	C _15_H _10_O _5_	269.0456	Negative	0.3–10	y = 0.104x + 0.0199	0.01/0.03	0.9998	81.55	4.07
**Hispidulin**	36.09	44.40	3.41	C _16_H _12_O _6_	301.0707	Positive	0.05–10	y = 0.02614x + 0.0003114	0.01/0.03	0.9993	98.36	2.91
**Caffeic acid phenethyl ester**	0.19	0.20	3.13	C _17_H _16_O _4_	283.0976	Negative	0.3–7	y = 0.255x + 0.0477	0.01/0.03	0.9964	94.42	3.66
**Chrysin**	2.22	1.47	3.24	C _15_H _10_O _4_	253.0506	Negative	0.05–7	y = 0.0964x − 0.0002622	0.01/0.03	0.999	87.92	2.36
**Acacetin**	3.75	4.37	3.98	C _16_H _12_O _5_	283.0612	Negative	0.05–7	y = 0.046x + 0.0001875	0.01/0.03	0.9995	87.52	3.55
**Dihydrocaffeic acid**	76.97	100.48	0.86	C _18_H _23_NO _7_	366.15473	Negative	0.5–10	y = 0.06102x − 0.00989	0.999	0.14/0.46	100.77	**1.05**
**6-OH-luteolin-7-** ***O*** **-glucoside**	33.91	24.64	2.99	C _21_H _20_O _12_	463.0882	Negative	0.5–10	y = 0.01482x − 0.001803	0.997	0.23/0.77	102.44	**3.66**
**6-O-Me-luteolin-7-** ***O*** **-glucoside**	175.75	123.73	2.58	C _21_H _20_O _12_	477.10449	Negative	0.5–10	y = 0.02636x − 0.00254	0.9982	0.18/0.61	101.21	**3.16**
**Chrysoeriol**	23.22	25.63	2.08	C _16_H _12_O _6_	299.05611	Negative	0.5–10	y = 0.1023x − 0.002224	0.9974	0.15/0.5	96.42	**2.55**
**Cirsilineol**	7.97	21.71	2.33		313.07227	Negative	0.5–10	y = 0.1389x + 0.02817	0.9955	0.22/0.72	96.17	**2.86**
**Apigenin 7-methylate**	0.50	0.60	2.94	C _15_H _12_O _5_	283.06149	Negative	0.5–10	y = 0.3563x	0.9949	0.16/0.54	97.1	**3.60**
**Sclareol**	2.91	1.43	3.96	C _20_H _36_O _2_	273.25745	Positive	0.5–10	y = 0.3233x + 0.0004172	0.9984	0.12/0.39	100.59	**4.85**
**Homogentisic acid**	1162.53	1664.52	4.35	C _8_H _8_O _4_	167.03498	Negative	0.5–10	y = 0.01076x − 0.002027	0.9987	0.13/0.45	100.76	5.33
**3,4-dihydroxy benzaldehyde**	49.04	51.19	3.79	C _7_H _6_O _3_	137.02442	Negative	0.5–10	y = 1.343x + 0.6441	0.9962	0.53/1.77	98.67	4.64
**2,5-dihydroxybenzoic acid**	30.38	45.71	4.77	C _7_H _6_O _4_	153.01933	Negative	0.5–10	y = 0.06114x − 0.0102	0.9985	0.17/0.57	100.23	5.84
**Salvianolic acid B**	5163.41	4893.41	6.50	C _36_H _30_O _16_	717.14611	Negative	1–10–	y = 0.0005342x − 0.0003522	0.9989	0.33/1.1	98.96	7.96
**Hispidulin 7-** ***O*** **-glucoside**	4586.70	3825.48	4.57	C _22_H _22_O _11_	461.10893	Negative	0.5–10	y = 0.0003758x − 0.00006441	0.9988	0.57/1.91	101.76	5.60
**Carnosic acid**	0.19	0.20	2.58	C _20_H _28_O _4_	331.19148	Negative	0.5–10	y = 0.342x + 0.0485	0.9984	0.21/0.69	100.14	3.16

R^2^: coefficient of determination; LOD/LOQ (mg/L): limit of detection/quantification.

## Figures and Tables

**Scheme:**
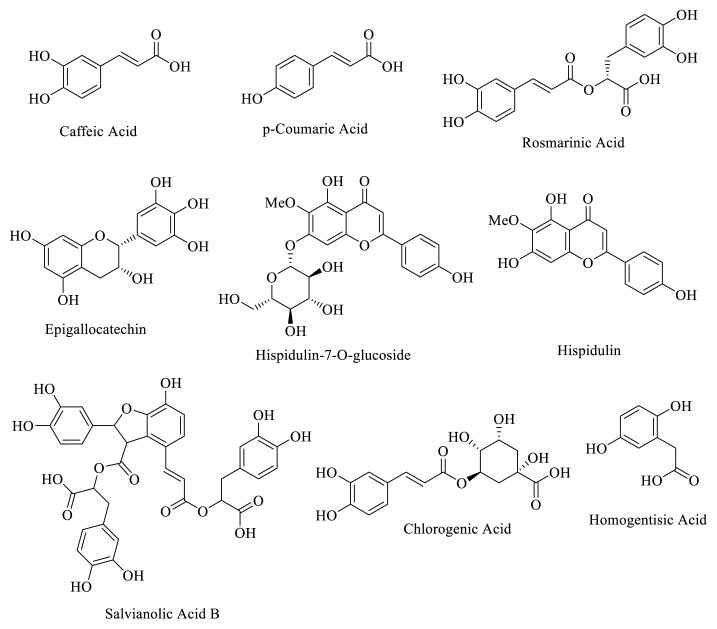
The chemical formula of docked compounds.

**Table 1:** Compounds detected in *Salvia* species in ethanol extract, infusion, and decoctions (g/kg extract).

Compounds	Ethanol		Infusion		Decoction	
	ST	SH	ST	SH	ST	SH
**Ascorbic acid**	393.50	598.48			273.18	333.73
**(-)-Epigallocatechin**	300.55	508.75	293.28	211.15	538.47	615.99
**(-)-Epigallocatechin gallate**			10.06	4.25	12.57	5.16
**Chlorogenic acid**	249.51	135.84	305.49	131.31	824.02	540.08
**Verbascoside**	161.64	146.72	11.29	10.95	41.49	38.89
**Orientin**	16.91	139.26	1.59	8.21		23.02
**Caffeic acid**	3784.07	4560.78	2453.24	1750.28	5446.51	6159.05
**Caffeine**		0.24				
**(+)-** ***trans*** ** Taxifolin**	59.73	88.01	1.71	2.94	5.90	6.11
**Luteolin-7-** ***O*** **-rutinoside**	815.81	932.03	87.63	121.03	244.06	267.58
**Vanillic acid**	146.93	400.84				
**Luteolin 7-** ***O*** **-glucoside**	512.52	615.14	28.23	34.76	119.20	111.90
***p*** **-Coumaric acid**	1173.23	1664.39				
**Hesperidin**	84.34	42.33	11.41	6.98		
**Rutin**	21.92	10.88		0.83	1.49	
**Rosmarinic acid**	58,912.33	59,015.24	30,283.78	21,426.51	47,396.59	48,091.43
**Hyperoside**	202.58	274.36	21.71	16.90	38.93	51.31
**Apigenin-7-** ***O*** **-glucoside**	312.33	271.89	9.62	7.78	33.30	25.67
**Nepetin-7-** ***O*** **-glucoside**	182.78	179.97	85.57	45.40	157.78	116.31
**Quercetin**	3.99	9.70	0.48	0.52	2.30	2.66
**Salicylic acid**	651.19	823.34	162.03	127.42	243.56	285.99
**Naringenin**	11.23	16.66	1.59	1.23	0.73	1.31
**Luteolin**	175.15	172.33	38.97	24.68	38.47	57.82
**Nepetin**	66.54	79.19	8.51	5.04	6.63	9.01
**Apigenin**	332.92	236.93	18.49	13.02	24.37	26.63
**Hispidulin**	258.04	377.60		24.29	36.09	44.40
**Caffeic acid phenethyl ester**	0.55	1.01	0.12	0.04	0.19	0.20
**Chrysin**	1.41	1.32	0.87	0.67	2.22	1.47
**Acacetin**	13.74	30.71	2.54	2.66	3.75	4.37
**Dihydrocaffeic acid**	21.53	35.14	75.90	72.94	76.97	100.48
**6-OH-luteolin-7-** ***O*** **-glucoside**	14.05	14.39	16.06	13.89	33.91	24.64
**6-** ***O*** **-Me-luteolin-7-** ***O*** **-glucoside**	130.37	89.43	98.37	52.50	175.75	123.73
**Apigenin 7-** ***O*** **-acylglucoside**	48.57	20.17				
**Luteolin-7-** ***O*** **-acylglucoside**			174.99	141.79		
**Chrysoeriol**	155.54	176.96	18.65	14.48	23.22	25.63
**Circilineol**	32.21	35.71			7.97	21.71
**Apigenin 7-methylate**	1.84	4.05	0.36	0.36	0.50	0.60
**Sclareol**	1.72	5.37	0.72	0.36	2.91	1.43
**Homogentisic acid**	929.78	1331.28	779.92	639.56	1162.53	1664.52
**3,4-dihydroxybenzaldehyde**	164.27	223.31	30.18	15.24	49.04	51.19
**2,5-dihydroxybenzoic acid**	33.03	30.95	20.87	23.81	30.38	45.71
**Salvianolic acid B**	14,911.62	13,111.55	3458.41	2785.28	5163.41	4893.41
**Hispidulin 7-O-glucoside**	26,517.26	26,709.56	1976.26	1796.51	4586.70	3825.48
**Pinocembrin**	0.39	0.34				
**Genkwanin**	0.04	1.72				
**Carnosic acid**	1.96	0.03	0.32	0.32	0.19	0.20

**Table 2:** Docking scores of selected compounds on TNF-α, iNOS, CA-I and CA-II.

Compounds	Docking scores (kcal/mol)			
	TNF - α (7KP9)	iNOS (4NOS)	hCA - I (1BZM)	hCA - II (1A42)
Caffeic acid	−5.944	**−11.395**	−6.390	**−8.047**
*p-*Coumaric acid	−5.657	−8.774	−6.013	−6.851
Rosmarinic acid	−10.952	−8.542	**−10.439**	**−8.557**
Salvianolic acid B	**−12.391**	**−17.547**	**−7.877**	**−14.312**
Hispidulin- 7- *O*- glucoside	**−11.473**	**−10.167**	−7.806	−7.365
Hispidulin	−8.947	−8.905	−6.149	−6.764
Homogentisic acid	−6.089	−9.664	−7.639	−7.784
Epigallocatechin	−11.243	−9.224	−7.142	−7.248
Chlorogenic acid	−10.976	−9.417	−7.611	−6.980
Aspirin *(reference)*	−5.695	−9.162	-	-
Celecoxib *(reference)*	−8.339	−5.376	-	-
Acetazolamide *(reference)*	-	-	−4.797	−5.690

## References

[1] Newman DJ, Cragg GM (2016). Natural Products as Sources of New Drugs from 1981 to 2014. Journal of Natural Products.

[2] Barnes J, Anderson LA, Phillipson JD (2007). Herbal medicines.

[3] Topcu G, Yücer R, Senol H, GV, Pavlov A (2018). Bioactive Constituents of Anatolian Salvia Species. Salvia Biotechnology.

[4] Tan N, Yazici-Tutunis S, Yesil Y, Demirci B, Tan E (2017). Antibacterial Activities and Composition of the Essential Oils of Salvia sericeo-tomentosa Varieties. Records of Natural Products.

[5] Celep F, Dogan M, Bagherpour S, Kahraman A (2009). A New Variety of Salvia sericeotomentosa (Lamiaceae) from South Anatolia, Turkey. Novon.

[6] Aydin SK, Ertas A, Boga M, Erol E, Toraman GOA (2021). Di-, and Triterpenoids Isolation and LC-MS Analysis of Salvia marashica Extracts with Bioactivity Studies. Records of Natural Products.

[7] Topcu G, Akdemir A, Kolak U, Ozturk M, Boga M (2020). Anticholinesterase and Antioxidant Activities of Natural Abietane Diterpenoids with Molecular Docking Studies. Current Alzheimer Research.

[8] Topcu G (2006). Bioactive triterpenoids from Salvia species. Journal of Natural Products.

[9] Ulubelen A, Sonmez U, Topcu G (1997). Diterpenoids from the roots of Salvia sclarea. Phytochemistry.

[10] Sen Utsukarci B, Gurdal B, Bilgin M, Satana D, Demirci B (2019). Biological Activities of Various Extracts from Salvia cassia Sam. ex Rech.f. and Chemical Composition of Its Most Active Extract. Records of Natural Products.

[11] Ulubelen A, Topcu G, Tan N (1995). Diterpenoids from salvia-heldrichiana. Phytochemistry.

[12] Scott A, Khan KM, Cook JL, Duronio V (2004). What is “inflammation”? Are we ready to move beyond Celsus?. British Journal of Sports Medicine.

[13] Zou YH, Zhao L, Xu YK, Bao JM, Liu X (2018). Anti-inflammatory sesquiterpenoids from the Traditional Chinese Medicine Salvia plebeia: Regulates pro-inflammatory mediators through inhibition of NF-kappa B and Erk1/2 signaling pathways in LPS-induced Raw264.7 cells. Journal of Ethnopharmacology.

[14] Minhas R, Bansal Y (2022). Inhibition of iNOS by Benzimidazole Derivatives: Synthesis, Docking, and Biological Evaluations. Medicinal Chemistry.

[15] Şenol H, Çelik Turgut G, Şen A, Sağlamtaş R, Tuncay S (2023). Synthesis of nitrogen-containing oleanolic acid derivatives as carbonic anhydrase and acetylcholinesterase inhibitors. Medicinal Chemistry Research.

[16] Saikia S, Bordoloi M (2019). Molecular Docking: Challenges, Advances and its Use in Drug Discovery Perspective. Curr Drug Targets.

[17] Şenol H, Ağgül AG, Atasoy S, Güzeldemirci NU (2023). Synthesis, characterization, molecular docking and in vitro anti-cancer activity studies of new and highly selective 1,2,3-triazole substituted 4-hydroxybenzohyrdazide derivatives. Journal of Molecular Structure.

[18] Cadirci E, Suleyman H, Gurbuz P, Uz AK, Guvenalp Z (2012). Anti-inflammatory effects of different extracts from three Salvia species. Turkish Journal of Biology.

[19] Margetts G, Kleidonas S, Zaibi NS, Zaibi MS, Edwards KD (2022). Evidence for anti-inflammatory effects and modulation of neurotransmitter metabolism by Salvia officinalis L. Bmc Complementary Medicine and Therapies.

[20] Zhang SY, Luo H, Sun SY, Zhang YT, Ma JQ (2022). Salvia miltiorrhiza Bge. (Danshen) for Inflammatory Bowel Disease: Clinical Evidence and Network Pharmacology-. Based Strategy for Developing Supplementary Medical Application Frontiers in Pharmacology.

[21] Ozer Z, Goren AC, Kilic T, Oncu M, Carikci S (2020). The phenolic contents, antioxidant and anticholinesterase activity of section Amaracus (Gled.) Vogel and Anatolicon Ietsw. of Origanum L. species. Arabian Journal of Chemistry.

[22] Tokalı FS, Şenol H, Bulut Ş, Hacıosmanoğlu-Aldoğan E (2023). Synthesis, characterization and molecular docking studies of highly selective new hydrazone derivatives of anthranilic acid and their ring closure analogue Quinazolin-4(3H)-ones against lung cancer cells A549. Journal of Molecular Structure.

[23] Şenol H, Çağman Z, Katmerlikaya TG, Tokalı FS (2023). New anthranilic acid hydrazones as fenamate isosteres: Synthesis, characterization, molecular docking, dynamics & in silico adme, in vitro anti-inflammatory and anti-cancer activity studies. Chemistry & Biodiversity.

[24] Luo CX, Zou L, Sun HJ, Peng JY, Gao C (2020). A Review of the Anti-Inflammatory Effects of Rosmarinic Acid on Inflammatory Diseases. Frontiers in Pharmacology.

[25] Ozturk M, Duru ME, Ince B, Harmandar M, Topcu G (2010). A new rapid spectrophotometric method to determine the rosmarinic acid level in plant extracts. Food Chemistry.

[26] Şenol H, Ağgül AG, Atasoy S (2023). Synthesis, characterization, molecular docking and in vitro biological studies of thiazolidin-4-one derivatives as anti-breast-cancer agents. ChemistrySelect.

[27] Tokalı FS, Taslimi P, Sadeghi M, Şenol H (2023). Synthesis and evaluation of quinazolin-4(3H)-one derivatives as multitarget metabolic enzyme inhibitors: A biochemistry-oriented drug design. ChemistrySelect.

